# Pressure Anesthesia Contra-Indicated

**Published:** 1917-01

**Authors:** 


### PRESSURE ANESTHESIA CONTRA-INDICATED.

The important contra-indication which seems to have been pretty generally overlooked is the one regarding the presence of a degree of infection in the pulp, a condition which most decidedly contra-indicates its employment. It is of extreme importance in this connection to bear clearly in mind the indisputable fact that pulp exposure invariably spells pulp infection, and that unless the exposure is of very recent occurrence, the painless removal of the pulp must be accomplished by methods other than cocain pressure-anesthesia. Consequently, we argue that the only cases in which the use of cocain pressure-anesthesia is indicated are those of ''fresh'' exposure, and those in which, because of prosthetic reasons, a normal pulp has to be sacrificed, and will have to be bared prior to the application of the anesthetic solution. With intra-osseous, infiltration and conductive methods of inducing local anesthesia which are at the disposal of every practitioner (and even arsenic in selected cases), there should be no room for cocain pressure-anesthesia except in the special cases above mentioned. The use of cocaine pressure-anesthesia, without regard to the presence of infection in the pulp, must be condemned as a dangerous empiricism. The forcing of blood contaminated by bacteria and their products, into the peri-apical structures in the endeavor to anesthetize the pulp, results in some of the peridental difficulties which dentists and physicians alike are endeavoring to prevent. *The field for cocain pressure-anesthesia is limited and its misapplication may result in a chronic focus of infection with all its attended complication.—Pacific Dental Gazette.*

